# Treatment of Cryptococcal Meningitis in KwaZulu-Natal, South Africa

**DOI:** 10.1371/journal.pone.0008630

**Published:** 2010-01-07

**Authors:** Josephine V. J. Lightowler, Graham S. Cooke, Portia Mutevedzi, Richard J. Lessells, Marie-Louise Newell, Martin Dedicoat

**Affiliations:** 1 Ngwelezane Hospital, Empangeni, KwaZulu-Natal, South Africa; 2 John Radcliffe Hospital, Oxford, United Kingdom; 3 Africa Centre for Health and Population Studies, University of KwaZulu-Natal, Mtubatuba, South Africa; 4 Department of Infectious Diseases, Imperial College London, London, United Kingdom; 5 UCL Institute of Child Health, London, United Kingdom; 6 University of Limpopo, Limpopo, South Africa; University of Minnesota, United States of America

## Abstract

**Background:**

Cryptococcal meningitis (CM) remains a leading cause of death for HIV-infected individuals in sub-Saharan Africa. Improved treatment strategies are needed if individuals are to benefit from the increasing availability of antiretroviral therapy. We investigated the factors associated with mortality in routine care in KwaZulu-Natal, South Africa.

**Methodology/Principal Findings:**

A prospective year long, single-center, consecutive case series of individuals diagnosed with cryptococcal meningitis 190 patients were diagnosed with culture positive cryptococcal meningitis, of whom 186 were included in the study. 52/186 (28.0%) patients died within 14 days of diagnosis and 60/186 (32.3%) had died by day 28. In multivariable cox regression analysis, focal neurology (aHR 11 95%C.I. 3.08–39.3, P<0.001), diastolic blood pressure <60 mmHg (aHR 2.37 95%C.I. 1.11–5.04, P = 0.025), concurrent treatment for tuberculosis (aHR 2.11 95%C.I. 1.02–4.35, P = 0.044) and use of fluconazole monotherapy (aHR 3.69 95% C.I. 1.74–7.85, P<0.001) were associated with increased mortality at 14 and 28 days.

**Conclusions:**

Even in a setting where amphotericin B is available, mortality from cryptococcal meningitis in this setting is high, particularly in the immediate period after diagnosis. This highlights the still unmet need not only for earlier diagnosis of HIV and timely access to treatment of opportunistic infections, but for better treatment strategies of cryptococcal meningitis.

## Introduction

Despite the increasingly widespread availability of highly active antiretroviral therapy (HAART) throughout sub-Saharan Africa, HIV remains the leading cause of death amongst adults in many populations, particularly in rural Southern Africa [1] Prior to the availability of antiretrovirals, cryptococcal meningitis (CM) accounted for a significant proportion of deaths in HIV infected individuals [2]–[4] and even with increasing availability of HAART, recent data suggests that CM may account for more deaths amongst HIV positive individuals in sub-Saharan Africa than tuberculosis [5].

For many individuals with HIV, their first presentation to health services is with a major opportunistic infection such as CM and optimal management in these individuals is crucial if they are to benefit fully from antiretroviral therapy. Data from different healthcare settings is valuable for the identification of factors associated with treatment outcome and understanding which interventions are necessary to improve survival. The clinical diagnosis and management of CM differ between clinical settings depending on resources available [6]. Availability of diagnostics, drugs and trained nursing and medical staff, as well as other broader health systems issues influencing timely access to healthcare are all likely to be relevant to outcomes.

We set out to understand the factors associated with outcome for patients with CM in a public sector hospital setting within northern KwaZulu-Natal, South Africa, where the prevalence of HIV infection reaches 20% or more among the general adult population.

## Methods

This study was carried out at Ngwelezane hospital, a 550 bed regional government hospital in Northern KwaZulu-Natal, South Africa. KwaZulu-Natal is the South African province worst affected by HIV with an estimated 1.5 million infected individuals [7]. The hospital offers both district and regional services. 440,000 people fall under the hospital's district catchment area, only patients from this area were included in the study as for patients referred from other hospitals it was not possible to get baseline information.

All patients diagnosed with cerebrospinal fluid (CSF) culture positive CM during 2007 were included in the study. Verbal informed consent was obtained from the patient or relatives for the collection of relevant data from the patient's chart.

The decision to initiate CM treatment was based on a clinical diagnosis supported by positive Indian Ink microscopy. Patients were treated according to local hospital protocol with amphotericin B (AmB) 0.7 mg/kg daily for 14 days when supplies were available. Liposomal formulations were not available. 1 litre of normal saline was given before each dose of AmB, further intravenous fluids were given at the discretion of the treating doctor. Where possible lumbar puncture was repeated on days 7 and 14. Additional lumbar punctures were carried out if the patient developed severe headache believed by the clinician to be due to CM. CSF pressure measurement was not routinely performed as appropriate equipment was usually not available. Patients who had renal impairment on admission (define as serum creatinine >220 umol/l) were treated with fluconazole 400 mg daily rather than AmB in line with South African treatment guidelines at the time of the study. If a patient's renal function improved following hydration, AmB was started at standard dosage.

All patients had full blood counts, electrolytes (U&E) and liver function tests performed prior to initiating therapy with AmB. U&E was repeated every 48 hours. Electrolyte abnormalities were corrected where possible. Where possible, cannulation sites were rotated every 72 hours to prevent thrombophlebitis. Patients developing renal impairment were given increased intravenous fluids. If a patient's creatinine rose to >220 umol/l AmB was stopped and fluconazole 400 mg daily started. If the patients renal function improved AmB was restarted. If the patient's renal function deteriorated again AmB was terminated for good and substituted with fluconazole 400 mg daily. Flucytosine was not available during the study period.

As a regional centre, the hospital has both CT and MRI scans available. There is an on-site intensive care unit (ICU), but medical patients are not routinely admitted to the unit and none of the patients in this study were admitted to ICU. HAART has been available locally through public sector provision since 2004. All patients of unknown HIV status were offered VCT when clinically well enough to consent, no patients were tested anonymously or without consent. All patients not on HAART were offered treatment under South African guidelines that take any WHO stage IV illness (in this case CM) as an eligibility criterion for initiating HAART. HAART was started only after 14 days of AmB treatment, either as an inpatient, at a chronic care facility, or most usually, at an outpatient ART clinic following discharge.

Data were collected by a physician (MD) using a standardized data collection form. All patients were reviewed by the clinical team daily, and study data was updated every 48 hours. Data were entered into an excel spread sheet and verified on a separate occasion. For survival analysis, patients were right censored at date of discharge or date of later follow-up visit, with the outcome being mortality. Person time was calculated as days from date presenting at the hospital, as opposed to onset of disease, to date of death or date of discharge or review for those still alive. Kaplan- Meier analysis was used to determine the overall time to death and mortality rate as well as 14 and 28 day mortality rates. Cox regression analysis with Breslow method for ties was used to assess variables that were independently associated with 14 day and 28 days mortality. STATA 10 (College Station, Texas, USA) was used for analysis.

## Results

Overall 92/186 (49.5%) individuals were male with a median age of 33.5 years (IQR 28–39.5). The median age for women was 30.5 years (IQR 27–36), significantly lower than for men (P = 0.019). Baseline characteristics are shown in Table 1.

**Table 1:** Description of 186 patients diagnosed with Cryptococcal meningitis, presenting at Ngwelezana hospital.

Variable	n		n for group	%	
sex	186	Male	92	49.5	
		female	94	50.5	
			Over all	male	female
age	186	Mean	33.8	34.6	33.04
		Median	32	33.5	30.5
		IQR	27–38	28–39.5	27–36
			n for group	%	
Case type	186	New	154	82.8	
		Retreatment	32	17.2	
Headache	186	No	29	15.6	
		yes	157	84.4	
Fever	186	No	153	82.3	
		yes	33	17.7	
Fits	186	no	171	91.9	
		yes	15	8.1	
Confusion	186	No	144	77.4	
		yes	42	22.6	
Vomiting	186	No	101	54.3	
		yes	85	45.7	
Neck pain	186	No	43	23.1	
		yes	143	76.9	
Zoster	186	no	181	97.3	
		yes	5	2.7	
Focal Neurology	186	no	181	97.3	
		Yes	5	2.7	
CD4 cells/ul	119	Mean	90.8		
		Median	46		
		IQR	17–100		
Systolic BP	175	Mean	115		
		Median	113		
		IQR	101–125		
		<100	39	22.3	
		Normal (100–140)	114	65.1	
		>140	22	12.6	
Diastolic BP	175	Mean	74.1		
		Median	72		
		IQR	63–87		
		<60	30	17.1	
		Normal (60–90)	113	64.6	
		>90	32	18.3	
Glasgow Coma		Normal (15)	147	86	
Score		Abnormal (<15)	24	14	
Tuberculosis		Never	84	45.2	
		past	47	25.3	
		Current	38	20.4	
		Past and current	17	9.14	
HIV status	186	Positive on admission	112	60.2	
		Positive in hospital	26	14	
		unknown	48	25.8	
On ART	186	No	159	85.5	
		yes	27	14.5	
Illness duration	174	Mean	13.1		
		Median	7		
		IQR	Apr-14		
Treatment given	186	Amphotericin	148	79.6	
		Fluconazole	28	15.1	
		Both	1	0.5	
		none	9	4.8	
Reason	164	None	139	84.8	
		Renal failure	25	15.2	

* P for differences in median age by sex = 0.019.

148/186 (79.6%) individuals received amphotericin B, 28/186 Fluconazole (15.1%) and one patient both treatments. In 25/28 (89.3%) patients receiving initial treatment with fluconazole, the treatment decision was based on the presence of renal failure. In 16 cases initially treated with amphotericin, patients were changed to fluconazole because of the development of renal failure. Other adverse events were uncommon, the most frequent side effect being hypokalaemia that developed in 7/186 (3.8%) Individuals.

Overall mortality rate was 2.13 deaths per 100 person days (95% CI 1.66–2.72). Mortality rates were higher in the first 14 days (2.54 per 100 person days, 95% C.I. 1.94–3.33) than the second 14 days (2.12 per 100 person years 95% C.I. 1.65–2.73). 52/186 (28.0%) patients died as in-patients within the first 14 days and 60/186 (32.3%) by 28 days (see Table 2). Data for overall outcomes are shown in Table 2.

**Table 2:** Period mortality rates in a cohort of 186 patients admitted in hospital due to cryptococcal meningitis.

Time		N	%	Exposure time in days	Mortality rate per 100 person days	95% C.I
Overall	Alive	123	66.13			
	Dead	63	33.87	2963	2.13	1.66–2.72
14 days	Alive	134	72.04			
	Dead	52	27.96	2047	2.54	1.94–3.33
28 days	Alive	126	67.74			
	Dead	60	32.26	2829	2.12	1.65–2.73

### Survival Analysis

**Figure 1:**
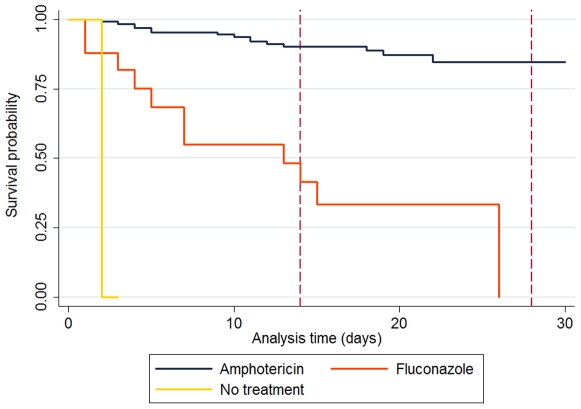
Kaplan-Meier time to death following diagnosis of cryptococcal meningitis and subsequent admission into hospital.

Kaplan Meier plots are shown in figure 1. Risks factors for 14 day and 28 day mortality are shown in Table 3. The final model had 175 patients for 14 day mortality and 171 for 28 day mortality. For 14 day mortality, 11 individuals had data missing with respect to diastolic blood pressure. The presence of focal neurological abnormality, diastolic hypotension, concurrent treatment for TB and the use of fluconazole rather than amphotericin, were associated with increased mortality at 14 and 28 days (Table 3).

**Table 3:** Risk factors for in-hospital mortality within 14 days and 28 days in patients diagnosed with cryptococcal meningitis.

Variable		14 day mortality	95% C.I.	*P*	28 day mortality	95% C.I.	*P*
Sex	Female	0.63	0.32–1.27	0.2	0.95	0.52–1.73	0.862
Age		0.99	0.96–1.03	0.72	0.99	0.97–1.03	0.843
Headache	No	1			N/S		
	Yes	0.58	0.28–1.19	0.137			
Focal neurology	No	1			1		
	Yes	11	3.08–39.28	**<0.001**	8.14	2.58–25.7	**<0.001**
CD4	>50	1			1		
	<50	0.7	0.29–1.66	0.413	1.02	0.48–2.20	0.951
	Missing	1.49	0.68–3.26	0.322	2.06	1.00–4.31	**0.05**
Diastolic BP	Normal	1			1		
	<60	2.37	1.11–5.04	**0.025**	2.34	1.18–4.63	**0.015**
	>90	1.64	0.68–3.99	0.272	1.1	0.49–2.44	0.824
	Missing	0.86	0.21–3.52	0.837	0.61	0.17–2.21	0.454
GCS	Normal (15)	1			N/S		
	Abnormal (<15)	2.05	0.91–4.59	0.081			
	Missing	1.57	0.64–3.83	0.323			
TB	Never	1			1		
	Past	1.83	0.79–4.23	0.158	1.88	0.88–4.01	0.101
	Current	2.11	1.02–4.35	**0.044**	2.2	1.13–4.30	**0.02**
	Past and current	0.92	0.26–3.29	0.903	0.62	0.18–2.11	0.445
Treatment	Amphotericin	1			1		
	Fluconazole	3.69	1.74–7.85	**0.001**	5.16	2.71–9.83	**<0.001**

## Discussion

Cryptococcal disease remains an important cause of mortality particularly in sub-Saharan Africa, despite the increasing availability of antiretrovirals. We describe a prospective series of individuals within a single health care setting operating within the public health sector of South Africa. The strengths of this data are that it is a large series, representative of outcomes in routine practice within South Africa.

A high proportion of the mortality observed in this unselected prospective case series was seen in the first 14 days. Unsurprisingly, the mortality rates observed are substantially higher than those in a western setting where mortality of 2.5–15% is more typical [8], [9] and have improved since the advent of HAART[10]. A significant number of individuals left hospital prior to completion of CM treatment and thus the true mortality might be higher than observed, though even if it were pessimistically assumed that all patients discharged alive died within the first 14 days after starting treatment, mortality rate would rise to only 31%. By comparison to a recent study from a centre in central Kampala, Uganda [11] mortality rates observed here were higher than seen in the post-HAART era raising important questions as to what factors are predictive of a poor outcome to treatment, though such differences could be explained by exclusion of comatose patients and those on fluconazole in that study.

The association of focal neurology with poorer outcome is perhaps not surprising, suggesting as it does the presence of more severe disease. The association between diastolic hypotension and increased mortality has not been described previously. High blood pressure can reflect raised intracranial pressure and might be expected to be associated with a poorer outcome. However, in this study, headache (also a feature of raised intracranial pressure) was associated with better outcome. Diastolic hypotension might reflect other concurrent infections (for example, bacterial infection) or metabolic disturbance (for example, hypoadrenalism) that may contribute to mortality. The association with concurrent tuberculosis treatment has not been described previously and is potentially specific to this population with very high rates of TB transmission. Assuming good adherence to medication, it is unlikely that these patients had active TB meningitis at the time of admission, although a small number of patients might have multi-drug resistant disease. Rifampicin can potentially reduce therapeutic levels of fluconazole but the effect on mortality was independent of treatment choice and most patients received amphotericin. Given the high burden of tuberculosis in local practice and limited diagnostics, there is a low threshold for initiation of empiric TB treatment in local practice and that it is possible that in some patients this delays the diagnosis of other conditions, including cryptococcal meningitis.

As results in table 3 demonstrate, there is clear evidence here for a poorer outcome in patients receiving fluconazole monotherapy rather than amphotericin. The majority of patients receiving fluconazole had co-existing renal failure which precluded the use of amphotericin (with liposomal formulations not available) and it is thus possible that the fluconazole-treated group over-represents individuals with severe disease. Given the co-linearity of The variables for choice of treatment and renal failure, it is not possible to separate these effects in analysis here, but a model including fluconazole monotherapy rather than renal failure provided a better fit to the data seen

Given the known superiority of AmB over fluconazole, a study of AmB in patients with renal failure could be justified from these data in resource-limited setting. In addition, studies using higher doses of fluconzaole will be helpful to improve the evidence base for non amphotericin based regimens[12]. Non-governmental guidelines issued during this study [13] recommend fluconazole at a higher dose of 800 mg/day and might offer additional benefit. Whether the addition of flucytosine to existing regimens would substantially impact mortality is unknown and the drug remains expensive. There is good evidence that the addition of flucytosine to AmB treatment is more rapidly fungicidal than AmB alone [14] though in a large RCT in a western setting [9] of 408 patients, no difference in mortality could be seen at two weeks. However, it remains possible that improved drug treatment could have more impact in a setting with higher background mortality where the relative impact of drug choice on mortality might be higher. Recent work the relationship between rate of fungal CSF clearance over the first 14 days of treatment and clinical outcome suggests a method by which different treatment combinations could be tested [15].

Perhaps surprisingly, there was no positive effect on outcome for patients established on HAART prior to diagnosis of CM. However, the duration of follow-up was probably not long enough to see the benefits of HAART seen elsewhere [16]. However, other data has suggested a potential benefit for HAART as early as two weeks [17], [18]. One of the limitations of this study was that it was not designed to study immune reconstitution inflammatory syndrome (IRIS). A proportion of those already on antiretrovirals might have experienced “unmasking IRIS” presenting early into treatment but data was not collected on duration of antiretroviral treatment to help understand this more. IRIS is undoubtedly a challenge for patients starting ART in the setting of CM [19]. Whilst ACTG 5164 [20], a strategic management trial largely recruiting in US centers with only a small number of CM patients, found no risk to early ART initiation, recent data from a larger study suggests a potential benefit to delaying the initiation of HAART in patients with CM [21]. However, early initiation of HAART following the diagnosis of CM was not common in this cohort and unlikely to have had a significant effect on mortality.

The context for our study was a regional referral hospital within the South African public sector. The facilities available lie between the extremes seen in developed countries and those in poorer (non-specialist) centres in sub-Saharan Africa. Drug treatment is available, with AmB the preferred first line option. Patients are usually managed by trained physicians but healthcare workers at all levels are in limited supply, drug and other medical supplies are inconsistent and supportive facilities (HDU,ICU), whilst theoretically available, are not routinely used for medically unwell patients. In well-resourced environments, it has become increasingly clear that the outcome for patients on ICU is not greatly different for those with HIV when compared to those without [22]. Data from this study can start to make an evidence based case for selecting those individuals who would benefit from more intensive support when it is available.

Detailed prevalence data from the nearby region [23] and experience of local antiretroviral programmes, suggests that the structure of the local population and relative prevalence of HIV between men and women, means more women both have HIV and access treatment. The similar proportions of men and women in this study therefore suggests that men within the local population are more likely than women to access care late and with WHO stage IV illness compared to what would be predicted. This cannot be fully substantiated with data from the wider immediate population, but is consistent with local experience. Late presentation is a key factor in improving outcome. With HAART widely available in many setting such as our own, all these deaths, and even disease presentations could be avoidable. The median duration of symptoms prior to presentation was 13 days in this study, very similar to that observed in Uganda [11]. Interventions that target earlier diagnosis and access to care are crucial if mortality is to be improved. In this regard, discussions within South Africa and elsewhere towards increasing the threshold at which individuals become eligible for antiretrovirals (in line with WHO recommendations) should be welcomed. In parallel to such efforts, studies looking to improve available treatments in resource poor settings should continue to be a priority.

## References

[1] Herbst AJ, Cooke GS, Barnighausen T, Kanykany A, Tanser F (2009). Adult mortality and antiretroviral treatment roll-out in rural KwaZulu-Natal, South Africa.. Bull World Health Organ.

[2] Okongo M, Morgan D, Mayanja B, Ross A, Whitworth J (1998). Causes of death in a rural, population-based human immunodeficiency virus type 1 (HIV-1) natural history cohort in Uganda.. Int J Epidemiol.

[3] French N, Gray K, Watera C, Nakiyingi J, Lugada E (2002). Cryptococcal infection in a cohort of HIV-1-infected Ugandan adults.. Aids.

[4] Corbett EL, Churchyard GJ, Charalambos S, Samb B, Moloi V (2002). Morbidity and mortality in South African gold miners: impact of untreated disease due to human immunodeficiency virus.. Clin Infect Dis.

[5] Park BJ, Wannemuehler KA, Marston BJ, Govender N, Pappas PG (2009). Estimation of the current global burden of cryptococcal meningitis among persons living with HIV/AIDS.. Aids.

[6] Jarvis JN, Harrison TS (2007). HIV-associated cryptococcal meningitis.. Aids.

[7] UNAIDS (2008). Report on the global AIDS epidemic..

[8] Chuck SL, Sande MA (1989). Infections with Cryptococcus neoformans in the acquired immunodeficiency syndrome.. N Engl J Med.

[9] van der Horst CM, Saag MS, Cloud GA, Hamill RJ, Graybill JR (1997). Treatment of cryptococcal meningitis associated with the acquired immunodeficiency syndrome. National Institute of Allergy and Infectious Diseases Mycoses Study Group and AIDS Clinical Trials Group.. N Engl J Med.

[10] Antinori S, Ridolfo A, Fasan M, Magni C, Galimberti L (2009). AIDS-associated cryptococcosis: a comparison of epidemiology, clinical features and outcome in the pre- and post-HAART eras. Experience of a single centre in Italy.. HIV Med.

[11] Kambugu A, Meya DB, Rhein J, O'Brien M, Janoff EN (2008). Outcomes of cryptococcal meningitis in Uganda before and after the availability of highly active antiretroviral therapy.. Clin Infect Dis.

[12] Longley N, Muzoora C, Taseera K, Mwesigye J, Rwebembera J (2008). Dose response effect of high-dose fluconazole for HIV-associated cryptococcal meningitis in southwestern Uganda.. Clin Infect Dis.

[13] McCarthy K, Meintjes G, Arthington-Skaggs E, Bicanic T, Cotton M (2007). Guidelines for the prevention, diagnosis and management of cryptococcal meningitis and disseminated cryptococcosis in HIV infected individuals.. Southern African Journal of HIV Medicine 25–35.

[14] Brouwer AE, Rajanuwong A, Chierakul W, Griffin GE, Larsen RA (2004). Combination antifungal therapies for HIV-associated cryptococcal meningitis: a randomised trial.. Lancet.

[15] Bicanic T, Muzoora C, Brouwer AE, Meintjes G, Longley N (2009). Independent association between rate of clearance of infection and clinical outcome of HIV-associated cryptococcal meningitis: analysis of a combined cohort of 262 patients.. Clin Infect Dis.

[16] Lortholary O, Poizat G, Zeller V, Neuville S, Boibieux A (2006). Long-term outcome of AIDS-associated cryptococcosis in the era of combination antiretroviral therapy.. Aids.

[17] Bicanic T, Meintjes G, Wood R, Hayes M, Rebe K (2007). Fungal burden, early fungicidal activity, and outcome in cryptococcal meningitis in antiretroviral-naive or antiretroviral-experienced patients treated with amphotericin B or fluconazole.. Clin Infect Dis.

[18] Bisson GP, Nthobatsong R, Thakur R, Lesetedi G, Vinekar K (2008). The use of HAART is associated with decreased risk of death during initial treatment of cryptococcal meningitis in adults in Botswana.. J Acquir Immune Defic Syndr.

[19] Lawn SD, Bekker LG, Myer L, Orrell C, Wood R (2005). Cryptococcocal immune reconstitution disease: a major cause of early mortality in a South African antiretroviral programme.. Aids.

[20] Zolopa A, Andersen J, Powderly W, Sanchez A, Sanne I (2009). Early antiretroviral therapy reduces AIDS progression/death in individuals with acute opportunistic infections: a multicenter randomized strategy trial.. PLoS One.

[21] Makadzange A, Ndhlovu C, Takarinda K, Reid M, Kurangwa M (2009). Early vs Delayed ART in the Treatment of Cryptococcal Meningtis in Africa.. CROI. Montreal.

[22] Dickson SJ, Batson S, Copas AJ, Edwards SG, Singer M (2007). Survival of HIV-infected patients in the intensive care unit in the era of highly active antiretroviral therapy.. Thorax.

[23] Barnighausen T, Tanser F, Gqwede Z, Mbizana C, Herbst K (2008). High HIV incidence in a community with high HIV prevalence in rural South Africa: findings from a prospective population-based study.. Aids.

